# EANM procedural guidelines for myocardial perfusion scintigraphy using cardiac-centered gamma cameras

**DOI:** 10.1186/s41824-019-0058-2

**Published:** 2019-07-02

**Authors:** Fabien Hyafil, Alessia Gimelli, Riemer H. J. A. Slart, Panagiotis Georgoulias, Christoph Rischpler, Mark Lubberink, Roberto Sciagra, Jan Bucerius, Denis Agostini, Hein J. Verberne, on behalf of the Cardiovascular Committee of the European Association of Nuclear Medicine (EANM)

**Affiliations:** 1 Department of Nuclear Medicine; Bichat University Hospital, Assistance Publique - Hôpitaux de Paris; Inserm UMR 1148, Paris Diderot-Paris 7 University, 46 rue Henri Huchard, 75018 Paris, France; 2 Fondazione Toscana/CNR Gabriele Monasterio, Pisa, Italy; 3 Medical Imaging Center, Department of Nuclear Medicine and Molecular Imaging, University Medical Center Groningen, Groningen, The Netherlands; 4 TechMed Centre, Department of Biomedical Photonic Imaging, University of Twente, Enschede, The Netherlands; 5 Department of Nuclear Medicine, University of Thessaly, University Hospital of Larissa, Larissa, Greece; 6 Department of Nuclear Medicine, University Hospital Essen, University of Duisburg-Essen, Essen, Germany; 7 Department of Medical Physics and PET Centre, Uppsala University Hospital, Uppsala, Sweden; 8 Nuclear Medicine Unit, Department of Experimental and Clinical Biomedical Sciences, University of Florence, Florence, Italy; 9 Department of Nuclear Medicine, Maastricht University Medical Center and Cardiovascular Research Institute Maastricht (CARIM), Maastricht University Medical Center, Maastricht, The Netherlands; 10 Department of Nuclear Medicine, University Hospital RWTH Aachen, Aachen, Germany; 11 Department of Nuclear Medicine, CHU Caen Normandy University, Caen, France; 12 Department of Radiology and Nuclear Medicine, Amsterdam UMC, University of Amsterdam, Amsterdam, The Netherlands

**Keywords:** Myocardial perfusion scintigraphy, Cardiac SPECT, CZT gamma camera, Procedural guidelines

## Abstract

An increasing number of Nuclear Medicine sites in Europe are using cardiac-centered gamma cameras for myocardial perfusion scintigraphy (MPS). Three cardiac-centered gamma cameras are currently the most frequently used in Europe: the D-SPECT (Spectrum Dynamics), the Alcyone (Discovery NM 530c and Discovery NM/CT 570c; General Electric Medical Systems), and the IQ-SPECT (Siemens Healthcare). The increased myocardial count sensitivity of these three cardiac-centered systems has allowed for a decrease in the activities of radiopharmaceuticals injected to patients for myocardial perfusion imaging and, consequently, radiation exposure of patients. When setting up protocols for MPS, the overall objective should be to maintain high diagnostic accuracy of MPS, while injecting the lowest activities reasonably achievable to reduce the level of radiation exposure of patient and staff. These guidelines aim at providing recommendations for acquisition protocols and image interpretation using cardiac-centered cameras. As each imaging system has specific design and features for image acquisition and analysis, these guidelines have been separated into three sections for each gamma camera system. These recommendations have been written by the members of the Cardiovascular Committee of EANM and were based on their own experience with each of these systems and on the existing literature.

## Preamble

The European Association of Nuclear Medicine (EANM) is a professional nonprofit medical association that facilitates communication worldwide among individuals pursuing clinical and research excellence in nuclear medicine. The EANM was founded in 1985.

These guidelines are intended to assist practitioners in providing appropriate nuclear medicine care for patients. They are not inflexible rules or requirements of practice and are not intended, nor should they be used, to establish a legal standard of care.

The ultimate judgment regarding the propriety of any specific procedure or course of action must be made by medical professionals taking into account the unique circumstances of each case. Thus, there is no implication that an approach differing from the guidelines, standing alone, is below the standard of care. To the contrary, a conscientious practitioner may responsibly adopt a course of action different from that set out in the guidelines when, in the reasonable judgment of the practitioner, such course of action is indicated by the condition of the patient, limitations of available resources or advances in knowledge or technology subsequent to publication of the guidelines.

The practice of medicine involves not only the science but also the art of dealing with the prevention, diagnosis, alleviation, and treatment of disease. The variety and complexity of human conditions make it impossible to always reach the most appropriate diagnosis or to predict with certainty a particular response to treatment. Therefore, it should be recognized that adherence to these guidelines will not ensure an accurate diagnosis or a successful outcome. All that should be expected is that the practitioner will follow a reasonable course of action based on current knowledge, available resources, and the needs of the patient to deliver effective and safe medical care. The sole purpose of these guidelines is to assist practitioners in achieving this objective.

These guidelines summarize the views of the Cardiovascular Committee of the EANM and reflect recommendations for which the EANM cannot be held responsible. The recommendations should be taken into context of good practice of nuclear medicine and do not substitute for national and international legal or regulatory provisions. The guidelines were brought to the attention of all other EANM Committees and to the National Societies of Nuclear Medicine. The comments and suggestions from the French and Israelian national societies are highly appreciated and have been considered for these guidelines.

## Introduction and technical overview

A growing number of nuclear medicine sites in Europe are now using a new generation of cardiac-centered gamma cameras for myocardial perfusion imaging. Three cardiac-centered gamma cameras are currently the most frequently used in Europe: the D-SPECT (Spectrum Dynamics), the Alcyone (General Electric Medical Systems), and the IQ-SPECT (Siemens Healthcare). The heart-centric method of collimation of these systems explains the enhanced count sensitivity, when compared with conventional Anger cameras. In addition, iterative reconstruction methods taking into account the collimation geometry have improved image contrast and decreased the level of noise (Erlandsson et al. 2009; Imbert et al. 2012). The tomographic count sensitivity is a critical parameter influencing both the acquisition time and the injected activity of tracer. This sensitivity is relatively low on conventional gamma cameras with only a few parts-per-million (10^−6^) of injected activities being detected within the myocardial area (Imbert et al. 2012; Verger et al. 2014). On Anger cameras, this fraction may be increased when using a collimator with convergent geometry such as the SMARTZOOM multifocal collimator system for IQ-SPECT focused on the heart. The IQ-SPECT orbit is centered on the heart instead of the gantry’s mechanical center, ensuring that the heart is always in the SMARTZOOM collimators’ magnification area. The count sensitivity is enhanced by a factor of two when using the IQ-SPECT technology instead of a conventional LEHR collimator (Imbert et al. 2012). In so-called “CZT cameras,” the conventional sodium/iodine (NaI) crystal used for the detection of gamma rays has been replaced by a cadmium-zinc-telluride (CZT) crystal. This crystal transforms directly the signal induced by gamma rays into electric impulses without the need for photo-detectors. The interaction of a gamma photon of 140 keV in CZT detectors produces approximately 30,000 electrons, 20-fold more than that produced in a conventional NaI crystal, improving the energy resolution by a factor of 2 compared with conventional Anger cameras. Manufacturers have taken advantage of these much thinner and more flexible CZT detectors to design gamma cameras dedicated to cardiac imaging offering a larger surface for signal detection, which is focused on the heart region. CZT gamma cameras provide a four- to sevenfold higher system sensitivity compared to NaI-based cameras (Imbert et al. 2012). Cardiac-centered CZT cameras are currently commercially available from two different vendors: the D-SPECT family from Spectrum Dynamics (D-SPECT, D-SPECT L, and D-SPECT Cardio) and cameras with the so-called Alcyone technology from GE Healthcare (Discovery NM 530c and Discovery NM/CT 570c). These cameras use the identical type of squared CZT crystals but differ regarding number of detectors, collimators, and reconstruction algorithms. Using a CZT camera equipped with heart-centric collimators, the tomographic count sensitivity is increased up to three- to fourfold with the Discovery NM530c camera and seven- to eightfold with the D-SPECT camera in comparison to conventional Anger cameras (Imbert et al. 2012). The geometry of the D-SPECT system with wide-angle parallel-hole collimators explains the higher increase in count sensitivity observed with this camera in comparison to the pinhole-based geometry of the Alcyone cameras. Thanks to the higher energy resolution of CZT in comparison to NaI crystals, the proportion of scatter signal in the images is decreased translating into an improvement in the contrast-to-noise ratio and spatial resolution of images from phantoms as well as in vivo SPECT acquired with CZT compared to conventional Anger cameras (Erlandsson et al. 2009; Imbert et al. 2012; Zoccarato et al. 2016; Ben-Haim et al. 2010). The pinhole-based geometry of the Alcyone system provides the highest increase in spatial resolution of images in comparison to the D-SPECT camera and to conventional gamma cameras (Imbert et al. 2012; Zoccarato et al. 2016; Imbert and Marie 2016) with full width at half maximum of punctual sources at the center of field of view measured at 6.7 mm with the Alcyone, 8.6 mm with the D-SPECT, and 15 mm for the IQ-SPECT system (Imbert et al. 2012).

The enhanced myocardial count sensitivity of these three cardiac-centered systems has allowed for a decrease in the activities of radiopharmaceuticals injected into patients for MPS translating into lower radiation exposure (Agostini et al. 2016). The use of ^99m^Technetium-labeled (^99m^Tc) perfusion tracers should be preferred in most clinical situations over ^201^Thallium (^201^Tl), as the level of radiation exposure for patients is significantly lower. Two ^99m^Tc-labeled perfusion tracers are commercially available: ^99m^Tc-2-methoxyisobutylisonitrile (MIBI) and ^99m^Tc-1,2-bis [bis (2-ethoxyethyl) phosphino]-ethane (Tetrofosmin). When setting up protocols for MPS, the overall objective should be to maintain high diagnostic accuracy of MPS, while injecting the lowest activities reasonably achievable to reduce the level of radiation exposure of patient and staff (ALARA principle). Nevertheless, the amount of activity of radiopharmaceutical to be injected into patients will depend on the organization of the Nuclear Medicine site for performing stress tests, the performance of the camera (sensitivity of the system), the perfusion tracer (^99m^Tc-labeled perfusion tracers vs.^201^Tl), the acquisition protocol (one-day vs. two-day), and the image quality required to maintain high diagnostic performance of MPS.

These guidelines aim at providing recommendations for the specific aspects related to cardiac acquisition protocols and image interpretation using cardiac-centered cameras. For the modalities on how to perform stress protocols and MPS acquisitions using conventional gamma cameras, the readers are referred to previously published EANM guidelines (Hesse et al. 2005; Verberne et al. 2015). These guidelines will provide the recommendations of the Cardiovascular Committee of EANM for acquisition protocols and current clinical evidence for MPS for each available cardiac-centered gamma camera system, namely the D-SPECT family (Spectrum Dynamics), the Alcyone-based cameras (General Electric Medical System), and the IQ-SPECT (Siemens Healthcare).

### Recommendations for injected activities of perfusion radiotracers

The increased sensitivity of cardiac-centered systems allows for a reduction in the activity of radiopharmaceutical injected into patients with similar scan time, in the duration of SPECT acquisitions with similar injected activities, or both (i.e., some reduction of injected activity and some reduction in scan time). When selecting the activity of perfusion radiotracer injected to patients, the reduction in activities should be prioritized following the as low as reasonably possible (ALARA) principle (Table 1). Nevertheless, image quality, in particular in obese patients, should be maintained to allow for accurate interpretation of MPS. In addition, the duration of SPECT acquisition should not last more than 15 min to limit motion artifacts during the acquisition. It is therefore recommended to adjust the injected activity to body weight, in particular in obese patients, and to decrease slightly the targeted count level for ultra-low dose protocols so that the average duration of acquisitions is not too long.

**Table 1:** Summary of optimal injected activities radiopharmaceutical and acquisition parameters for each cardiac-centered gamma cameras and acquisition protocol

Gamma cameras	D-SPECT	Alcyone	IQ.SPECT	Conventional Anger camera
^99m^Technetium-labeled tracers (usual protocols)				
Selection of estimated total myocardial counts (scout view)	0.7–1.3 million counts	0.7–1.4 million counts	NA	NA
Injected activities				
One-day stress/rest	Stress, 2.5–3.5 MBq/kg (total IA, 150–300 MBq) Rest, 7.5–10.5 MBq/kg (total IA, 450–900 MBq)	Stress, 2.5–3.5 MBq/kg (total IA, 150–300 MBq) Rest, 7.5–10.5 MBq/kg (total IA, 450–900 MBq)	Stress, 2.5–3.5 MBq/kg (total IA, 150–300 MBq) Rest, 7.5–10.5 MBq/kg (total IA, 450–900 MBq)	Stress, 4 MBq/kg 250–400 MBq (total IA) Rest, 12 MBq/lg 750–1200 MBq (total IA)
Two-day stress/rest	Stress or rest, 3–5 MBq/kg (total IA, 180–500 MBq)	Stress or rest, 3–5 MBq/kg (total IA, 180–500 MBq)	Stress or rest, 3–5 MBq/kg (total IA, 180–500 MBq)	Stress or rest, 4–7 MBq/kg (total IA, 300–600 MBq)
Viability	3–5 MBq/kg at rest (total IA: 180–500 MBq)	3–5 MBq/kg (total IA: 180–500 MBq)	3–5 MBq/kg (total IA: 180–500 MBq)	Stress or rest, 4–7 MBq/kg (total IA, 300–600 MBq)
^99m^Tc-labeled tracers (ultra-low dose protocols)				
Selection of estimated total myocardial counts (scout view)	0.5–0.8 million counts	0.7–0.9 million counts	NA	NA
Injected activities				
One-day stress/rest	Stress, 120 MBq Rest, 360 MBq	Stress, 120 MBq Rest, 360 MBq	Stress, 100 MBq Rest, 300 MBq	NA
^201^Thallium				
Estimated total myocardial counts (scout view)	0.5–0.8 million counts	1.0 million counts	NA	NA
Injected activities				
Stress/redistribution	Stress, 0.5–1.5 MBq/kg (total IA, 50–90 MBq) (± injection of 37 MBq for redistribution)	Stress, 0.5–1.5 MBq/kg (total IA, 50–90 MBq) (± injection of 37 MBq for redistribution)	Stress, 1.0–1.5 MBq/kg (total IA, 74–111 MBq) (± injection of 37 MBq for redistribution)	Stress, 1.0–1.5 MBq/kg (total IA, 74–111 MBq) (± injection of 37 MBq for redistribution)
Viability	0.5–1.5 MBq/kg at rest (total IA, 50–90 MBq)	0.5–1.5 MBq/kg at rest (total IA, 50–90 MBq)	1.0–1.5 MBq/kg at rest (total IA, 50–90 MBq)	Rest: 1.0–1.5 MBq/kg 74–111 MBq at rest (total IA)

### Protocols with ^99m^Tc-radiolabeled perfusion tracers

The reduction in activities injected to patients imaged with cardiac-centered gamma cameras results in syringes with small volumes, in particular, if the ^99m^Tc generator delivers an eluate with high activity. As ^99m^Tc-labeled perfusion tracers are “sticky,” a significant and variable amount of tracer may adhere to the inner part of the syringe and may thus not be injected into the patient. While this activity is negligible for protocols with conventional gamma cameras, the “true” activity injected into patients might be significantly reduced in syringes containing very small volumes according to ultra-low dose protocols resulting into long acquisition times and poor image quality. The residual activity in syringes should thus be monitored carefully with ultra-low dose protocols to have an accurate estimation of the true activity injected into patients. A dilution of the radiopharmaceutical after radiolabelling and before injection allows for an increase in the volumes of tracer injected into patients and may help to reduce the proportion of activity remaining in the syringe.

#### Two-day protocol

The average activity injected into patients should be in the range between 3 MBq/kg and 5 MBq/kg of ^99m^Tc-labeled perfusion tracer for stress and rest studies.

#### One-day protocol

For the first acquisition (stress or rest) , the injected activity should be in the range between 2.5–3.5 MBq/kg of ^99m^Tc-labeled perfusion tracer (minimal activity of 150 MBq—maximal activity of 300 MBq) and, for the second acquisition (stress or rest), in the range between 7.5–10.5 MBq/kg of 191 ^99m^Tc-labeled perfusion tracer (maximal activity of 900 MBq). Stress-first protocols should be preferred for one-day protocols because, in case of normal stress MPS, the second injection can be avoided resulting into a significant reduction of the radiation exposure of patients.

#### Viability

The activity injected into patients should be in the range between 3 MBq/kg and 5 MBq/kg of ^99m^Tc-labeled perfusion tracer at rest after administration of nitrates.

#### Ultra-low dose protocols

Ultra-low dose protocols with injection of fixed activities between 100 and 120 MBq provide also good image quality in non-obese patients (Einstein et al. 2014; Perrin et al. 2015; Songy et al. 2018). These protocols are particularly relevant for the screening of non-obese patients with low likelihood of CAD with radiation exposure of less than 2 milli-Sieverts (mSv) for stress-only acquisitions. Nevertheless, a compromise should be found between the decrease in activities injected to patients and the duration of SPECT acquisitions and image quality so that the diagnostic performance of MPS remain preserved using this ultra-low dose protocols.

### Protocols with ^201^Tl

The myocardial extraction fraction of ^201^Tl is two to three times higher than with ^99m^Tc-labeled perfusion tracers (Verger et al. 2014). The use of ^201^Tl can be interesting for the precise quantification of myocardial blood flow with SPECT using dynamic acquisitions. In addition, the redistribution of ^201^Tl in the myocardium allows for a more sensitive detection of viability, in particular in myocardial territory with chronic occlusion of coronary arteries. The higher tomographic sensitivity of cardiac-centered cameras allows for a reduction of injected activities of ^201^Tl of about 30% with preserved high image quality (Kincl et al. 2016; Songy et al. 2012). Nevertheless, the radiation exposure of patients for the acquisition of MPS with ^201^Tl is 2 to 3 times higher than with ^99m^Tc-labeled agents.

#### Stress-rest protocol

The recommended injected activity is 0.5–1.0 MBq/kg of ^201^Tl at stress for the D-SPECT and the Alcyone, and 1.0–1.5 MBq/kg of ^201^Tl at stress for the IQ-SPECT. An additional injection of 37 MBq of ^201^Tl might be injected at rest, 3 h after the stress, to improve the quality of redistribution images.

#### Viability protocol

The recommended injected activity is 0.5–1.0 MBq/kg of ^201^Tl at rest for the D-SPECT and the Alcyone, and 1.0–1.5 MBq/kg of ^201^Tl at rest for the IQ-SPECT.

### ^201^Tl/^99m^Tc protocols

The recommended injected activities for stress are between 0.5 and 1.0 MBq/kg of ^201^Tl and 2.5–3.5 MBq/kg of ^99m^Tc-labeled perfusion tracer for rest. Both acquisitions can be performed immediately after injection of the radiotracer and allow for stress/rest acquisitions in less than an hour (Berman et al. 2009; Barone-Rochette et al. 2018). This protocol offers the advantage of decreasing the prevalence of extra-cardiac signal on MPS at stress and at rest but is associated with higher radiation exposure of patients in comparison to previous protocols. This protocol can also be applied to shorten the duration of regular ^201^Tl protocols by replacing the redistribution acquisition performed 4 h after the injection by a rest acquisition after injection of ^99m^Tc. Attenuation artifacts and the intensity of extra-cardiac signal can vary between MPS obtained with each radiotracer. Comparison of stress and rest MPS acquired with dual isotope protocol should thus be performed very carefully.

## Image acquisition

### D-SPECT (Spectrum dynamics)

#### Design of the camera

The camera consists of 6 or 9 pixelated detector columns (depending on the camera version) arranged in a curved configuration that encloses the left side of the patient’s chest. The camera is equipped with tungsten parallel-hole collimators that are shorter and have larger square holes compared to standard lead parallel-hole LEHR collimators. Behind each collimator column, there is an array of 2.46 × 2.46 mm, 5-mm-thick CZT crystals (16 × 64 pixels, 40 mm × 160 mm) (Gambhir et al. 2009). Each detector column can rotate along its long axis up to 110° independently and focuses on the heart region. The larger holes of these collimators improve the number of counts detected originating from the myocardium in comparison to conventional gamma cameras with LEHR collimators. Specific reconstruction algorithms that modelize the particular geometry of the wide-angle collimator have been implemented on this camera and allows for an improvement in spatial resolution and image contrast (Erlandsson et al. 2009).

#### Image acquisition

##### Patient positioning

**Fig. 1:**
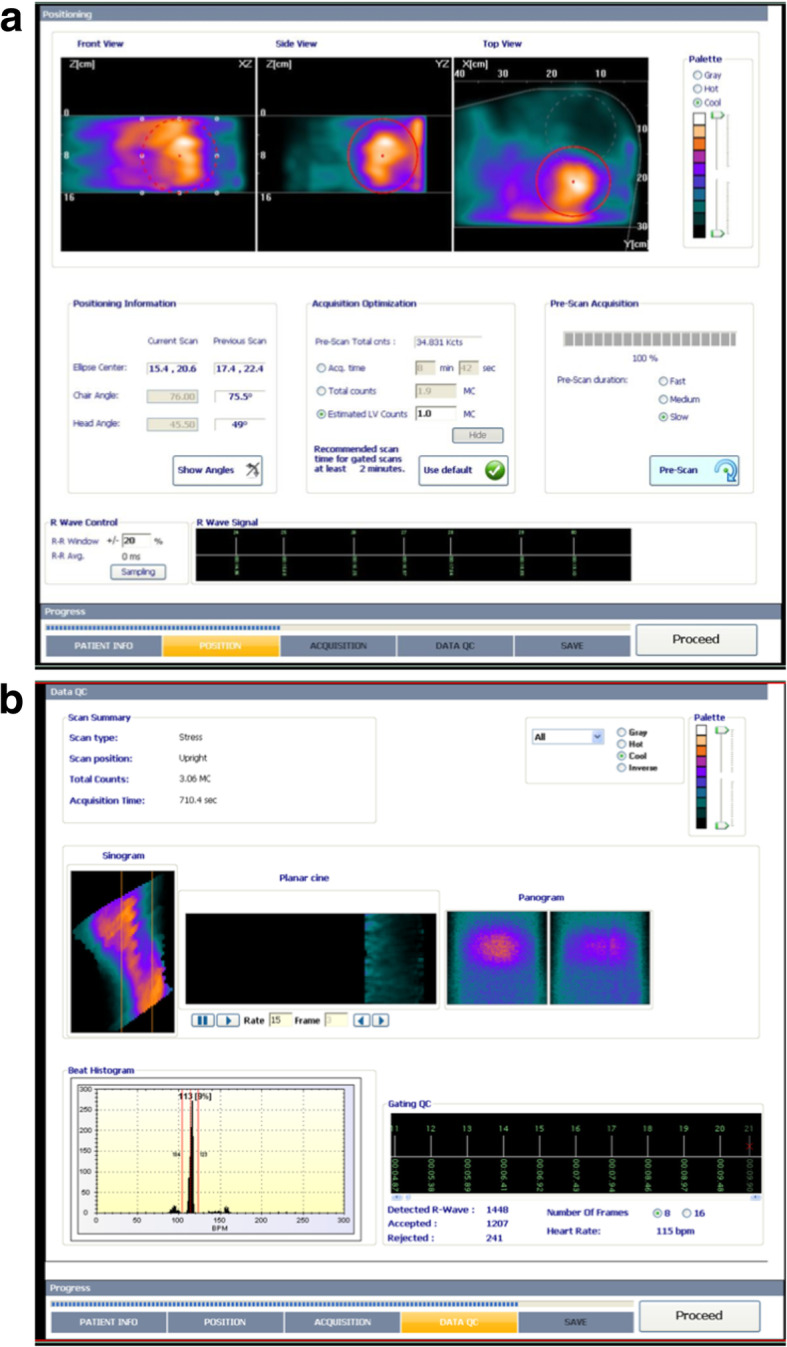
Example of quality control screens of MPS acquisitions with the D-SPECT camera. **a** Before starting the tomographic acquisition, a scout view is acquired that lasts 20–40 s to confirm that the detector arm is well positioned in the vertical axis and is close enough from the heart. The images located in the first row show that the detectors are well positioned in the vertical axis and include the whole heart in the field of view (left and middle images). Nevertheless, the heart (red circle, right image) is too far from the optimal position (circle with gray dotted line). Acquisition with the heart in this position may result in poor image quality. Efforts should be made to bring the detector arm closer from the chest and the heart of the patient. The duration of this low-dose stress acquisition after injection of a ^99m^Tc-labeled perfusion tracer was calculated at 8 min 42 s to reach an estimated LV myocardial count set at 1 million in the region of interest placed on the cardiac region on the scout view. **b** After the acquisition, a sinogram allows for the identification of patient movements in the horizontal axis and two panograms (for the two parts of the acquisitions separated by a movement of all the detectors in the arm) for the detection of movements in the vertical axis. Note the presence of patient movements in the vertical axis during the second part of the acquisition. In addition, the histogram shows all R-R intervals during the acquisition. Note the presence of abnormal short and long R-R intervals corresponding to extrasystolic and post-extrasystolic cardiac cycles. At the end of the acquisition, abnormal R-R intervals can be excluded from the gated reconstruction using the two red lines

The patient is placed on a dedicated seat associated to the camera in semi-supine position. The C-shaped arm containing the detectors is then approached progressively towards the chest of the patient. The left arm of the patient is placed on the upper edge of the camera arm containing the detectors. The camera arm containing the detectors should be placed as close to the chest as possible and to his left side, as the spatial resolution and image quality is decreasing with increasing distances between the heart and detectors. In obese patients with a large belly, inclining the seat can help to bring the arm with detectors closer to the chest of the patient. After placing the patient, a pre-scan of 30–60 s with a low spatial resolution is acquired to visualize the location of the cardiac signal (Fig. 1). This acquisition ensures that the seat of the patient is placed at the right height to include the whole heart in the “sweet spot” of the field of view. If not, the seat and the arm containing the detectors should be moved until the cardiac region is entirely included in the pre-scan and placed close enough of the detectors. Care should be taken to position the patient with similar settings for stress and rest acquisitions in order to have comparable acquisition conditions and artifacts on both sets of images.

##### Acquisition protocols

Acquisitions can be performed early after the injection of tracer (5–10 min pi), even with ^99m^Tc-labeled perfusion tracers. The high-energy resolution of CZT crystals allows for a decrease of the scatter signal from the liver and the digestive tract on MPS and preserved image quality on early acquisitions (Meyer and Weinmann 2017). Nevertheless, if high tracer uptake is present close to the heart (in the liver or digestive tract) with ^99m^Tc-labeled perfusion tracers, the acquisition should be repeated 30–40 min later after ingestion of a lipid-rich meal and cold water to stimulate the excretion of tracer from the liver and stimulate the digestive peristaltism.

The duration of SPECT acquisitions can either be of fixed length or adjusted to the counts measured in the myocardium that are estimated by placing a circular region of interest placed in the cardiac area on the scout view. The activity-based approach should be preferred as it allows for the adjustment of the duration of the SPECT acquisition to injected activity, myocardial extraction of the radiotracer, and tissue attenuation. Nevertheless, attention should be given to ensure that the ROI is placed exclusively on the heart and does not include extra-cardiac activities (liver, digestive tract) that might otherwise reduce inappropriately the duration of SPECT acquisitions and result in poor image quality. This count-based approach permits also to acquire stress and rest acquisitions with 1-day protocol images in similar conditions, even though the activities injected at stress and at rest differ by a factor of 3. The usual targeted activities selected for total myocardial counts are in the range between 0.7 and 1.3 million counts for MPS acquisitions with ^99m^Tc-labeled perfusion tracers and 0.6 and 0.8 million counts for MPS acquisitions with ^201^Tl. The selection of the total number of myocardial counts for SPECT acquisition on the D-SPECT camera results from a compromise between injected activities, mean duration of SPECT acquisitions, and image quality.

The tomographic acquisition on the D-SPECT camera is composed of two parts of equal duration. During the first part of the acquisition, the detectors rotate inside the arm of the camera and cover the cardiac region. At half of the acquisition time, the detectors are moved automatically in the horizontal direction for a short distance which provides additional angles of projections for the detectors. Consequently, if the acquisition is stopped prematurely on this camera, no image can be reconstructed and the whole acquisition needs to be repeated. For patients who are not able to stay immobile during the SPECT acquisition, it is therefore recommended to shorten the total length of SPECT acquisitions in order to get interpretable images.

##### Quality controls

Quality control of the camera should be performed on a daily basis with a cobalt rod source positioned in a dedicated arm that is attached to the machine to confirm that all detectors are functioning correctly and provide a homogeneous signal before starting any acquisition. At the end of each acquisition, the quality of SPECT data should be checked systematically using the dedicated tool available on the workstation (Fig. 1). A sinogram and two panograms are displayed at the end of acquisition as part of the quality control process. Image acquisition is composed of two parts of equal duration with a small horizontal translation of all detectors in the middle of the acquisition (Allie et al. 2016). The two sweeps of all detectors are resumed into two panograms and a sinogram. The sinogram allows for the identification of patient movement in the horizontal axis and panograms are used for the detection of motion in the vertical axis. The absence of any significant vertical or horizontal movements during the acquisitions should be verified on the sinogram and two panograms available at the end of the acquisition, as they might result in artifacts on the image and degrade image quality of the MPS study.

In a window, the histograms of R-R intervals during the acquisition are visualized. As acquisitions are saved in a list mode, the software provides the possibility to select with two bars the interval of R-R values for the reconstruction of gated images. This tool is particularly useful to select only regular cycles in patients with supra-ventricular or ventricular extrasystoles. Of note, the exclusion of a significant proportion of R-R cycles may result in a degradation of the quality of gated images, in particular, if more than 30% of the cycles are excluded. If arrhythmia is identified before starting SPECT acquisitions, 30% or 50% time can be added to the duration estimated on the scout image in order to maintain image quality of gated images after exclusion of the arrhythmic cycles at the end of the acquisition.

A view of the thorax is reconstructed and allows to visualize the heart and the structures surrounding the heart. This image is particularly useful to evaluate the intensity of extra-cardiac digestive or liver signal and the presence of increased lung uptake and to identify structures (diaphragm, breast, cell phone, metal objects) that might cause attenuation artifacts on MPS. Even though this view looks similar to the aspect of SPECT projections from conventional gamma cameras, it is already a 3D-reconstruction from the projection of the detectors and cannot be used to identify patient movements or to correct images for motion.

##### Image reconstruction

MPS images are reconstructed on the workstation of the D-SPECT camera using a dedicated algorithm that takes into consideration the geometry of the detecting system, the distance from the detectors, and the shape of the heart. Reconstruction is performed in 2 steps. In the first step with 3 iterations, the left ventricular (LV) region and orientation are determined and LV counts are calculated. Four further iterations are subsequently performed (Nakazato et al. 2013). The reconstruction algorithm is based on the maximum-likelihood expectation maximization method with resolution recovery (4–7 iterations and 32 subsets) and additional kernel convolution smoothing resulting into transaxial images (Gambhir et al. 2009). A Gaussian post-reconstruction filter as well as a proprietary normalizing post-reconstruction filter are used. No attenuation correction and no scatter correction are applied. Transaxial images are automatically reoriented into short-axis and vertical and horizontal long-axis slices using the quantitative perfusion SPECT software (QPS, Cedars-Sinai Medical Center). Neither motion correction, nor attenuation correction is currently available for the D-SPECT. However, the possibility to correct for attenuation using an externally acquired CT is currently in the process of implementation.

The choice of reconstruction parameters is relatively limited for users. The influence of the cardiac model in the reconstruction process can be decreased in reconstructions. This can be useful when the level of noise is high or in women with small hearts and virtual LV cavity. In addition, dedicated filter parameters that smoothen and decrease the level of noise on SPECT images might be considered for patients imaged with ultra-low dose protocols (Perrin et al. 2015). After reconstruction of SPECT acquisitions, vertical long-axis views are obtained that can be exported into other workstations for post-processing of MPS. For gated reconstructions, the recommended number of temporal frames is 16. For MPS with low-dose ^201^Tl, the number of temporal frames might be decreased to 8 to limit the level of noise on gated images.

##### Pitfalls and artifacts

As SPECT acquisitions are acquired in semi-supine position and the gamma camera is not associated to CT, attenuation correction of the SPECT images is usually not performed (the vendor is currently implementing the possibility to correct for attenuation using an externally acquired CT). In very obese patients, the most frequent limitation to perform the SPECT acquisition is the size of the inner circumference of the camera arm containing the detectors. It is therefore recommended to confirm before the injection of any radiotracer that the arm of the detector can be moved close enough to the thorax of the patient to allow for SPECT acquisitions. If the heart of the patients is placed too far from the detectors, image quality is degraded. Repeating acquisition after optimizing the positioning of the patient and the detector arm can help to improve image quality significantly. On the D-SPECT camera, attenuation artifacts caused by the diaphragm usually predominate in the apical segments of the inferior and infero lateral walls because of the semi-supine position of the patient during acquisitions (Allie et al. 2016). In addition, breast attenuation artifacts are frequent in the apical segments. Rim-filter artifacts can also occur in the presence of intense tracer uptake at the same level as the inferior wall and result into an artefactual low signal in the inferior wall. In this situation, the MPS acquisition should be repeated 45 min later after giving cold water to the patient to stimulate digestive peristaltism so that the signal present in the digestive tract moves to another location.

#### Clinical validation

##### Comparison of MPS with D-SPECT and conventional gamma cameras

The D-SPECT camera offers comparable diagnostic performance to that of conventional SPECT cameras on a per-patient basis, while achieving superior image quality and faster image acquisition owing to improved count sensitivity and image contrast. High agreement rates were found between images acquired with the D-SPECT camera and Anger camera for the classification of abnormal MPS (Gambhir et al. 2009). In a multi-centric study, Sharir et al. (Sharir et al. 2010) confirmed that the extent of stress and rest total perfusion deficit correlated linearly between D-SPECT and Anger cameras with good concordance in the evaluation of three vascular territories (> 90% agreement). The extent of myocardial ischemia was slightly but significantly larger on the D-SPECT compared with conventional SPECT. The value for ejection fraction and end-diastolic volume acquired on each camera were strongly correlated (*r* = 0.89 and 0.97, respectively). Verger et al. (2013) confirmed in a prospective multicenter study good correlations between the extent of stress defects (*r* = 0.86) and infarction area (*r* = 0.80) measured on the D-SPECT in comparison to Anger cameras, with slightly lower correlation for the extent of myocardial ischemia (*r* = 0.72). In the MultIcenter nucLear Low-dose Imaging at a milliSIEVERT (MILLISIEVERT) study (Einstein et al. 2014), 101 patients were imaged with the D-SPECT at rest with an ultra-low dose protocol (130 MBq of ^99m^Tc-labeled perfusion tracer; effective dose of 1.3 mSv) and using a conventional Anger camera with a standard low-dose protocol (average activity of 278 MBq of ^99m^Tc-labeled perfusion tracer). Overall image quality was superior with the D-SPECT in comparison to the conventional gamma camera with twice as many studies graded excellent quality; correlations between MPS acquired with each gamma camera was high for summed rest score (SRS; *r* = 0.87), total perfusion deficit (TPD; *r* = 0.91), and LV ejection fraction (LVEF; *r* = 0.88). High image quality could also be reached in 118 obese patients (60 of them were morbidly obese) with the D-SPECT. None of the patients had a non-diagnostic study. In obese patients, the upright position was associated with a lower rate of equivocal studies than the supine position and should be preferred (Ben-Haim et al. 2014).

##### Diagnostic performance of the D-SPECT vs. conventional gamma cameras

Nakazato et al. (2010) evaluated the diagnostic accuracy of MPS with the D-SPECT for the detection of coronary artery disease (CAD) in comparison with invasive coronary angiography. They first validated normalcy maps for the D-SPECT in patients with low likelihood of CAD (< 15%). Thresholds to define abnormal MPS based on automated analysis were then set for a TPD ≥ 5% in upright or supine acquisitions and ≥ 3% when both upright and supine acquisitions were combined. For per-vessel analysis, a threshold ≥ 2% in each coronary artery territory were considered as abnormal. Using this methodology, they reported in a series of 56 patients sensitivities of 91%, 88%, and 94% and specificities of 59%, 73%, and 86% for the detection of significant coronary stenosis on per-patient basis for upright, supine, or combined acquisitions, respectively, and sensitivities of 67%, 66%, and 69% and specificities of 91%, 94%, and 97% on a per-vessel basis. In another multi-centric study (Neill et al. 2013), the diagnostic performance of the D-SPECT has been evaluated in 50 patients with coronary angiography as gold standard. In this study, a global summed stress score (SSS) ≥ 3 or coronary territorial SSS ≥ 2 was considered as abnormal by visual analysis and a global TPD > 5% and coronary territorial TPD ≥ 3% defined as abnormal by automated analysis. The overall accuracy of MPS with D-SPECT was significantly higher than MPS acquired with conventional SPECT by visual assessment (90% vs. 76%, respectively) but similar between both gamma cameras using automated analysis (80% vs. 84%, respectively). Among 2845 patients evaluated with a low-dose protocol with ^99m^Tc-radiolabeled tracers (120 MBq at stress and 360 MBq at rest), Perrin et al. (2015) evaluated the diagnostic performance of SPECT-MPS with the D-SPECT in a sub-group of 149 patients who were referred for invasive coronary angiography. Sensitivity, specificity, and accuracy for the presence of coronary stenosis > 50% were 88%, 61%, and 80%, respectively. In addition, normalcy rate was 97% in patients with low likelihood of CAD who did not undergo coronary angiography.

##### Prognostic value

Only a few studies have assessed the prognostic value of MPS acquired with the D-SPECT. Xu et al. (2011) confirmed in a cohort of 1613 patients who underwent MPS acquired with the D-SPECT that the severity of the total perfusion deficit on MPS is associated with an increase in all-cause mortality in a similar way to what has been described for MPS acquired with conventional gamma cameras. Patients with normal MPS have low risk of cardiovascular events (< 2% annualized rate of non-fatal myocardial infarction, cardiac death). The annual rate of cardiovascular events increased with the extent of the perfusion defect, from 1.9% for small defects to 3.0% for moderate defects, up to 5.3% for large defects.

### The Alcyone camera (GEMS)

#### Design of the camera

The design of the GE camera with Alcyone technology is based on stationary multi-pinhole collimation system (Buechel et al. 2010). Each pinhole has an effective aperture diameter of only 5.1 mm. The design of the system offers a predominant increase in spatial resolution over count sensitivity (Imbert et al. 2012). Nevertheless, the sensitivity is also higher in comparison to acquisitions performed with LEHR collimators on conventional gamma cameras with NaI crystals thanks to the large surface of the 19 pinhole-detector blocks focused on the cardiac region (Imbert et al. 2012). The stationary array simultaneously acquires all the views necessary for tomographic reconstruction, saving the time required by conventional cameras for acquisitions while rotating around the subject. All views simultaneously focus on the heart to maximize the efficiency of cardiac imaging. To fit the multiple views, the image is reduced in size by means of pinhole collimation, matching the miniaturization to the improved intrinsic pixel resolution of the detectors. This allows for the detector surface to be maximized increasing system efficiency. The pinhole geometry has several advantages. The reduction in pinhole sensitivity with increasing distance significantly diminishes the contribution of background organs and tissues to the cardiac data, facilitating reliable 3-dimensional iterative reconstruction. In addition, the oblique angles of incidence also improve the already higher intrinsic superior energy resolution of CZT compared to NaI crystals. A phantom study (Imbert et al. 2012) comparing the sensitivity of scanners from different vendors found that the Discovery NM 530 yielded substantially higher count sensitivity over standard SPECT (460 vs. 130 counts s^−1^ MBq^−1^). Similar results were documented when assessing myocardial counts normalized to injected activities in humans for the Alcyone technology and standard SPECT (i.e., 5.6 ± 1.4 and 0.6 ± 0.1 counts s^−1^ MBq^−1^, respectively). The central spatial resolution of the Discovery NM 530c was measured 6.7 mm compared to 15.3 mm with standard SPECT, also in accordance with the analysis of the sharpness of myocardial contours on human images (in cm^−1^, 1.02 ± 0.17 and 0.65 ± 0.06, respectively). These data document a dramatic enhancement in image quality mainly because of a lower proportion of Compton photons within the acquisition energy window. Moreover, CZT image quality was further improved by the development of a dedicated three-dimensional iterative reconstruction algorithm, based on maximum-likelihood expectation maximization (MLEM), which corrected for the loss in spatial resolution due to line response function of the collimator (Hudson and Larkin 1994). Clinical studies confirmed that this CZT camera allowed for a more than fivefold reduction in scan time and provided clinical information equivalent to conventional standard SPECT MPS (Buechel et al. 2010; Fiechter et al. 2011).

#### Image acquisition

##### Patient positioning

On cameras with Alcyone technology, patients are usually imaged in the supine position with arms placed over their head without any detector or collimator motion. Nishiyama et al. (2014) showed that prone imaging on the Alcyone camera can provide perfusion images of similar quality as the ones obtained in supine position, even though the distance between the detectors and the chest wall is increased in this position and the table supporting the patient can create some attenuation of the signal. Prone imaging can help to identify attenuation artifacts that have different positions on supine and prone acquisitions and thus to reduce the rate of false-positive studies.

##### Acquisition protocols

The duration of SPECT acquisitions can either be a fixed duration or adjusted to the activity measured in the myocardium that is estimated by placing a circular region of interest placed in the cardiac area on the scout view. The choice of the total number of myocardial counts for SPECT acquisition on CZT-GE camera results from a compromise between injected activities, mean duration of CZT scan acquisitions, and image quality. The protocol selected for a particular study should be tailored to the patient and to the clinical indication. No single protocol is optimal for every patient, and nuclear cardiology laboratories should strive to implement patient-centered imaging rather than performing the same protocol for each patient. This includes selecting an appropriate protocol and choosing administered activities that are appropriate for the patient’s habitus, i.e., weight-based dosing (Gimelli et al. 2018).

The targeted estimated total number of myocardial counts is usually set between 0.7 and 1.4 million counts for low-dose MPS acquisitions and between 1.2 and 1.8 million counts for high-dose MPS acquisitions (1-day protocols).

For ^201^Tl, acquisitions can be performed 5–10 min after the injection at stress and at rest. For ^99m^Tc-sestamibi or ^99m^Tc-tetrofosmin, acquisitions at rest or after pharmacological test are usually performed between 15 to 45 min after injection in order to allow for clearance of the radiopharmaceutical from the liver and to reduce background signal in the digestive tract. In patients stressed with physical exercise, acquisitions can be started 15 min after injection because the myocardial extraction of ^99m^Tc-labeled radiopharmaceutical is increased by exercise and the background signal in the digestive tract is usually reduced. In presence of extra-cardiac activity that degrades the quality of myocardial perfusion images, the acquisition should be repeated 45 min after ingestion of a lipid-rich meal and cold sparkling water that help decrease the intensity of the liver and digestive background signal.

##### Quality controls

**Fig. 2:**
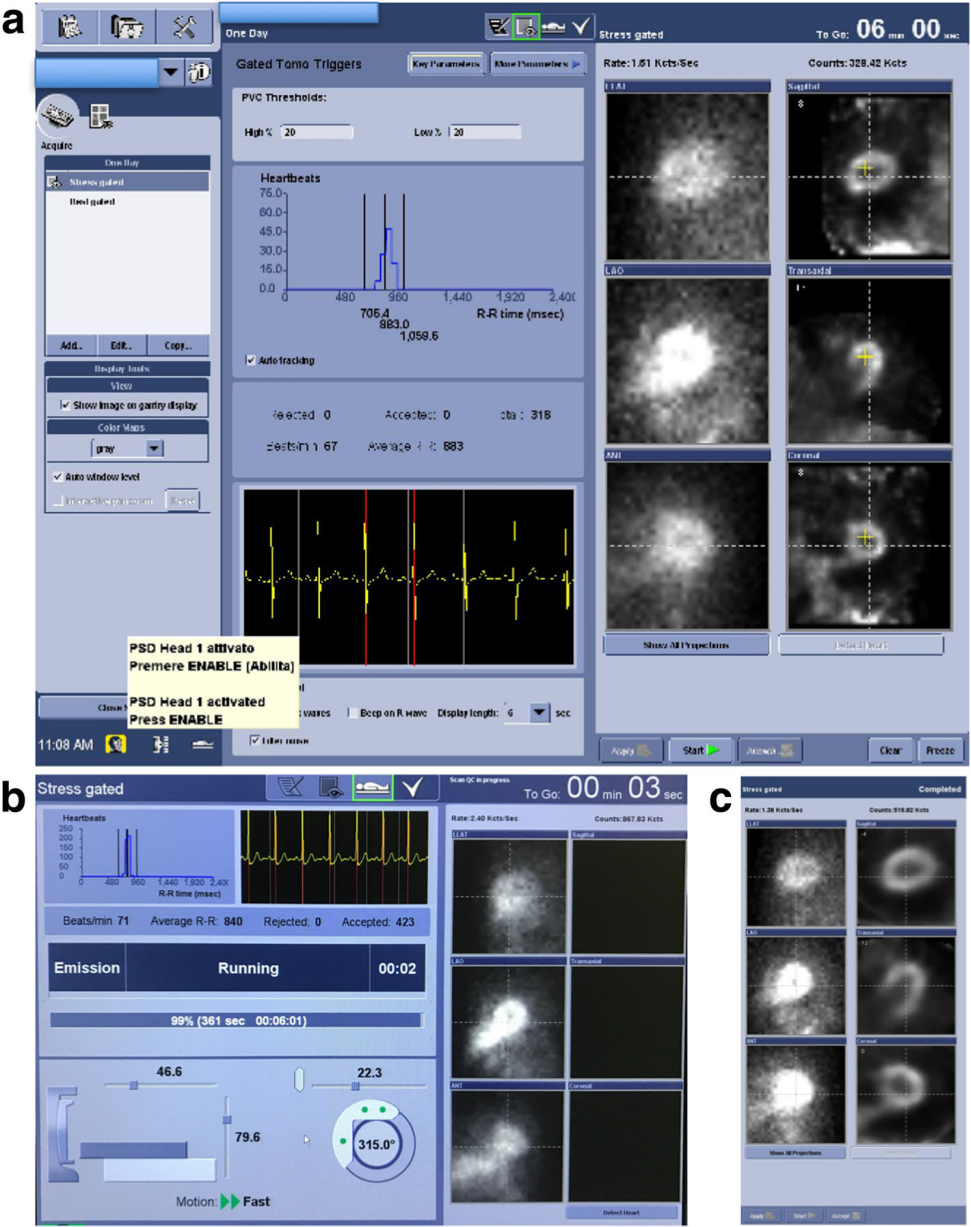
Example of quality control screens of MPS acquisitions with the GE camera using the Alcyone technology. **a** Before starting the tomographic acquisition, a scout view is acquired that lasts 20–40 s to confirm that the patient is well positioned in the vertical axis. The images located in the first row shows that the detectors is well positioned in the three axes and includes the whole heart in the field of view. The yellow cross (right images, in the three axes) should be placed the closest to the center of the heart. The duration of this low-dose stress acquisition after injection of a ^99m^Tc-labeled perfusion tracer was calculated at 6 min to reach an estimated LV myocardial count set at 1 million in the region of interest placed on the cardiac region on the scout view. **b** During the acquisition, a histogram shows all R-R intervals included in the acquisition. Note the presence of abnormal short and long R-R intervals corresponding to extrasystolic and post-extrasystolic cardiac cycles. At the end of the acquisition, abnormal R-R intervals can be excluded from the gated reconstruction. The proportion of accepted cardiac cycles should be sufficient to preserve image quality of gated images and obtain reliable values for LVEF. **c** At the end of the acquisition, on the right of the screen, the final quality of the three transaxial axes should be evaluated

The quality of SPECT acquisitions should be checked systematically on the dedicated tool of the workstation (Scan QC; Fig. 2). The scan quality control provides two options for performing quality control on slice data, SPECT QC with either organ and/or body views and Gated QC. Scan QC is necessary to view Alcyone stress and rest projections and slices, and the output displays gridlines indicating the center of the system field of view (FOV). The last step is the use of the contours that superimposes the threshold contours from the top scan slices on the bottom slices, for the correct assessment of myocardium position alignment.

The Gated QC screen displays average heart rate, accepted and rejected beats, curves depicting total counts before and gated bin normalization, heart rate during acquisition and R-R interval distribution.

Beat acceptance window is defined using two limits computed by applying a low and a high percentage to the “average” beat duration. Representative R-R time before acquisition should be computed before starting the actual acquisition. R-R intervals acceptance window size definition can be fixed by the user, but its actual center and width changes at run time with detected beats R-R interval. Physicians can decide to use time-mode acquisition (time/bin fixed at the beginning of acquisition, time_per_bin = Representative R-R time before acquisition) or phase-mode acquisition (time/bin changes for each accepted beat, time_per_bin = current R-R time). Time and phase modes are equivalent as long as the heart rate is stable throughout the scan. As phase mode tends to blur systolic and early diastolic phenomena at low and moderate heart rates, time mode should be preferred. At high heart rates or if just the visualization of wall motion is required, phase mode may be preferred. Patients with severe arrhythmia do not usually provide interpretable gated scans.

##### Image reconstruction

All images are acquired with a 32 × 32 matrix and a 20% energy window centered at the 140 keV photopeak of ^99m^Tc. List mode files are acquired and stored. Images should be reconstructed on a standard workstation (Xeleris II or higher; GE Healthcare, Haifa, Israel) using a dedicated iterative algorithm. All studies should be reconstructed using a standard iterative algorithm with ordered-subset expectation maximization with 50 iterations, without resolution recovery, or attenuation correction. A Butterworth post-processing filter (frequency 0.37, order 7) is applied to the reconstructed slices. The tomographic studies are re-projected into 60 planar projections to emulate a standard SPECT layout.

Gated stress and rest images are reconstructed in 16 frames and analyzed using the commercially available software. In patients with inadequate border detection, manual editing should be performed.

Alcyone technology is less sensitive to patient motion than regular SPECT cameras. All projections are acquired simultaneously avoiding inconsistency between different views, increasing sensitivity, and resulting in shorter duration of acquisitions. The approach for motion detection in GE’s CZT camera can be summed up in 5 steps: list data are binned into dynamic views (1 s for respiratory gating and 5 s for patient motion); data are reconstructed into 5 dynamic images from 5 central pinholes. An ellipsoid mask on dynamic images is created and finally, *x*,*y*,*z* coordinates of myocardium center of mass are derived for each time bin and automatically corrected if necessary. A dedicated tool may be available for attenuation correction: attenuation correction QC. A customization parameter allows images to contain either the unmasked left ventricle or the left ventricle masked according to left ventricle-based contours. In 2010, Herzog (Fiechter et al. 2011) evaluated the interest of attenuation correction of MPS acquired with the Discovery NM 530 camera. Segmental tracer uptake correlated strongly with attenuation-corrected MPS obtained from a conventional SPECT camera, and clinical agreement was excellent. Nevertheless, most Alcyone cameras are acquired as stand-alone systems and do not have an integrated computed tomography (CT) able to provide an attenuation map (DePuey 2012). Esteves et al. (2014) demonstrated the feasibility of attenuation correction of MPS using an attenuation map acquired on an external CT and found that attenuation correction of MPS resulted into a higher specificity without a loss in sensitivity for the detection of CAD. Caobelli et al. (2016) confirmed that CT-based AC using the Alcyone camera improves diagnostic accuracy in a similar way to Anger cameras. The effects of attenuation correction of MPS were most prominent in the RCA territory and, to a lesser degree, in the LCX territory but did not have any significant effect in the LAD territory. The use of AC of MPS on the Alcyone camera allows for better estimation of the presence and extent of perfusion defects, in particular in myocardial regions subject to important tissue attenuation, and helps to decrease the rate of false-positive studies.

##### Pitfalls and artifacts

The first demonstration of the origin of artifacts deriving from Alcyone technology has been published in 2014 by Liu et al. (2015). An anthropomorphic torso phantom and water bags to simulate breasts were used to evaluate artifacts arising from soft tissue attenuation. The study confirmed that Alcyone technology camera has better photon sensitivity, higher spatial resolution, and superior image quality than the conventional Anger camera (Imbert and Marie 2016; Takahashi et al. 2013). However, the sharpness and contrast-to-noise ratio of MPS are degraded in presence of important tissue attenuation, which explains why the Alcyone camera does not perform so well in very obese patients (Fiechter et al. 2012). Oddstig et al. (2018) compared the localization, extent, and importance of attenuation artifacts between a GE camera with Alcyone technology vs. a conventional gamma camera and found that attenuation artifacts were shifted counter-clockwise from the inferolateral to the lateral wall and were less intense with the Alcyone than with a conventional gamma camera. Thus, it is important that physicians interpreting MPS images are aware of these differences in attenuation patterns when interpreting non-attenuation-corrected images on the Alcyone camera. In doubtful situations, the use of attenuation correction based on CT (Nkoulou et al. 2011; Herzog et al. 2010; Mouden et al. 2012) can help to discriminate between true perfusion defects and attenuation artifacts.

#### Clinical validation

##### Comparison of MPS with Alcyone and conventional gamma cameras

In the study of Esteves et al. (2009), 168 patients underwent a 1-day ^99m^Tc-tetrofosmin rest/stress imaging protocol and were imaged both the GE camera with Alcyone technology and a conventional dual-detector SPECT gamma camera. Rest and stress acquisition times in patients with the same injected activities were 4 and 2 min for the GE camera with Alcyone technology and 14 and 12 min for the conventional SPECT gamma camera. Agreement for presence or absence of myocardial perfusion defects on a per-patient analysis between the Alcyone and conventional gamma cameras was 91.9% and 92.5%, respectively. Correlation coefficients of rest and stress left ventricular ejection fractions were 0.87 (*p* < 0.01) and 0.90 (p < 0.01). Buechel et al. (2010) found similar results in 75 consecutive patients imaged with a 1-day ^99m^Tc-tetrofosmin adenosine stress or rest imaging protocol. Conventional SPECT was acquired for 15 min for both stress and rest and compared with 3-min stress and 2-min rest acquisitions on the Alcyone camera. There was an excellent clinical agreement between the Alcyone and conventional gamma cameras on per-patient (96%) and on per-vessel territory basis (96%), also allowing for more than a fivefold reduction in scan time while providing clinical results equivalent to conventional camera. In addition, ventricular volumes and LVEF calculated on gated MPS acquired with the CZT camera correlated well with the values measured on cardiac MRI despite a small underestimation of the LV volumes with SPECT (Giorgetti et al. 2013). Image quality may be degraded in obese patients because the heart is often located at the border of the field of view of the camera resulting in relevant tissue attenuation. Fiechter et al. (2012) reported poor diagnostic performance in morbidly obese patients, with 81% non-diagnostic images. CT-based attenuation correction of MPS allowed for a reduction in the rate of non-diagnostic images down to 55% in this cohort. Kincl et al. (2016) tested the feasibility of an ultra-low-dose ^201^Tl protocol (injected activity reduced to 0.5 MBq/kg of ^201^Tl) using GE camera with Alcyone technology in 124 patients. Using 10-min gated acquisitions in the supine position image quality, image quality was preserved even in obese patients and radiation exposure of patients was significantly reduced (4–5 mSv).

##### Diagnostic performance of the Alcyone vs. conventional gamma cameras

The diagnostic performance of MPS for the detection of significant stenosis on invasive angiography was compared in 34 patients imaged both with GE’s CZT camera and with a conventional gamma camera. MPS with the CZT camera allowed for the detection of a higher number of vessels with obstructive CAD than with conventional SPECT, with a preserved level of diagnostic confidence on a per-patient basis (Gimelli et al. 2011). Moreover, the CZT camera identified with higher sensitivity the presence of perfusion defects in the territories of the left circumflex and right coronary artery territories in comparison to conventional gamma camera, resulting in a better identification of patients with multivessel CAD (Gimelli et al. 2017; Nudi et al. 2017). The diagnostic performance of low-dose MPS with a camera with the Alcyone technology and standard-dose MPS with conventional gamma camera was also compared in a group of 208 patients who underwent MPS and invasive coronary angiography and 76 low-risk patients. One-day stress-first MPS using the Alcyone technology and automated quantitative analysis provided high diagnostic value, similar to standard-dose MPS, and with 50% radiation reduction for stress-rest acquisitions (6.9 ± 1.1 vs. 11.7 ± 0.4 mSv) (Sharir et al. 2016). Using a dual isotope protocol (^201^Tl for stress, ^99m^Tc-labeled perfusion tracer for rest) in 54 patients referred for coronary angiography, sensitivity, specificity, and diagnostic accuracy were measured at 93%, 69%, and 81% for the detection of coronary stenosis > 50% with FFR < 0.8 (Barone-Rochette et al. 2018). The good diagnostic performance of MPS using the Alcyone camera for the diagnostic of significant coronary stenosis on invasive angiography has now been validated in a total of 1500 patients with sensitivities in the range between 77 and 95% and specificities in the range between 66 and 93%. Only one study reported a specificity of 37% that was likely caused by a referral bias of patients recruited in the study. Nevertheless, tissue attenuation and high tracer uptake in the adjacent bowel can affect the detection of perfusion defects in the inferolateral wall with this camera. In this situation, the combination of supine and prone acquisitions (Goto et al. 2014) helps to improve the specificity for the detection of coronary stenosis > 75% (93% vs. 72%) with any significant deterioration in sensitivity (68% vs. 82%).

##### Prognostic value

The prognostic value of MPS with the Alcyone camera was shown to be similar for the prediction of cardiovascular events as for values observed with MPS using conventional gamma cameras (Chowdhury et al. 2014; Oldan et al. 2016; Yokota et al. 2016). Yokota et al. (2016) have compared the incidence of major cardiac events in 1288 patients with normal stress-only CZT MPS and 362 patients with normal conventional SPECT and have demonstrated a comparable prognostic value with an incidence of 1.5% per year in the CZT group compared with 2% per year in the conventional group. The same group (Engbers et al. 2017) confirmed in a cohort of 4057 patients with suspected CAD that the annual event rate increased with the extent of abnormality on MPS, from 0.6% in patients with a normal study, to 2.8% in patients with small ischemic perfusion defects up to 4.3% in patients with moderate or large ischemic perfusion defects. In a group of 1288 patients with suspected CAD and imaged with ultra-low-dose MPS (150 MBq of ^99m^Tc-labeled perfusion tracer), the annual rate of cardiovascular event was measured at 0.5% after a 3-year follow-up for MPS classified as normal (Songy et al. 2018). In a group of 1128 patients imaged either with conventional MPS and 865 patients imaged with the GE’s CZT camera, the prediction of myocardial infarction or death within 2 years was similar between the two systems (Oldan et al. 2016). Of note, patients with high body mass index (BMI) were excluded from this study. Finally, Songy et al. (2018) showed in a cohort of 1400 patients that a normal MPS acquired using an ultra-low dose protocol (1.8 MBg/kg of ^99m^Tc-labeled perfusion tracer; estimated radiation exposure of patients of 1–2 mSv) in association with an exercise test was predictive of a low risk of cardiovascular events (annualized rates of cardiac events: 0.55%) after a mean follow-up of 39 months.

### IQ-SPECT

#### Design of the camera

IQ-SPECT cardiac imaging is based on a multifocal collimator system called SMARTZOOM. The system can be installed on Symbia Siemens cameras but is not supported on Symbia E and Symbia Evo Excel. SMARTZOOM collimators center on the heart, collecting up to 4 times more counts than LEHR collimators. These collimators magnify the heart while still capturing counts from the entire field of view. IQ-SPECT orbit is centered on the heart instead of the gantry’s mechanical center, ensuring that the heart is always in the SMARTZOOM collimators’ magnification area. Thus, the system is able to reduce acquisition time from approximately 20 min to approximately 4–5 min with the same patient dose. Although presented as optional by the manufacturer, attenuation correction (AC) using a CT scan (CTAC) with 2 to 16 detector rows depending on the exact model of Symbia camera is an additional and essential feature for IQ-SPECT.

#### Image acquisition

##### Patient positioning

**Fig. 3:**
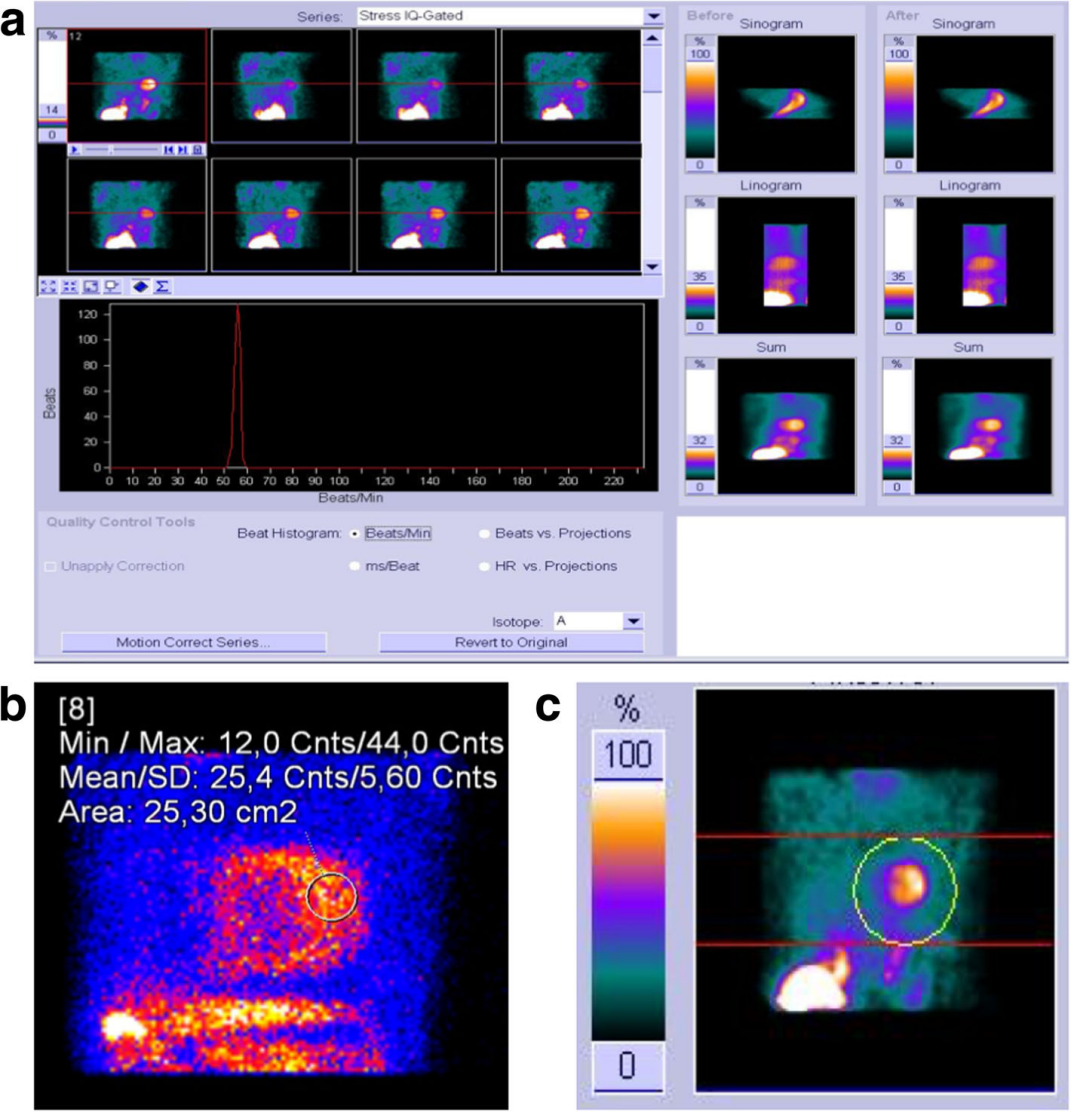
Example of quality control screens of MPS acquisitions with IQ-SPECT. **a** Before starting the tomographic acquisition, the entire patient set-up adds just one additional step to identify the position of the heart on the touch screen patient positioning monitor. The patient is placed on the bed in either supine or prone position, arms up, and moved under the nuclear detectors until the heart is approximately centered in the axial direction. The center of the projection of the heart on each detector is marked on the patient positioning monitor, allowing for the calculation of the location of the patient’s heart in 3-dimensional space. This will become the center of the cardio-centric orbit. The acquisition can then be started. The best way to immediately assess the quality of an IQ-SPECT study data is to load the raw projection series into the *syngo* Viewing tab. The projection data from a patient that has been positioned correctly will show a magnified heart at the center of every image as in the above example labeled Raw Projection Series. **b** There is a simple method to determine whether sufficient counts have been collected to produce an acceptable result in the reconstructed images. Load the projection data into the *syngo* Viewing tab and advance through the images to view 18 as in the example image below. This is the projection which contains the LAO view of the heart. Under the tool’s drop-down menu choose Circle or Freehand. Draw an ROI over the lateral wall as shown in the image below. Image statistics within the ROI will be calculated. It is important that the mean counts in the ROI over the lateral wall be at least 9 counts. **c** Acquisitions acquired with SMARTZOOM collimators can only be corrected for motion using the dedicated automatic motion correction tool and mask method. The operator should first review the data and determine if motion correction is required. One important factor in successful motion correction is the placement of the mask. In cases of extreme motion, it is best to try and re-image the patient

Factors influencing patient position include camera/gantry design, minimization of artifacts, and patient comfort. The supine position with the arms raised above the head is routinely used for IQ-SPECT imaging. For cardiac CT, supine positioning is standard. Appropriate table centering within the gantry is important to allow for proper function of angular *z*-axis tube current modulation. Both gated and non-gated acquisitions of ^99m^Tc and ^201^Tl are supported. The entire patient set-up adds just one additional step to identify the position of the heart on the touch screen patient positioning monitor. The patient is placed on the bed in either a supine or prone position and moved under the gamma-detectors until the heart is approximately centered in the axial direction (Fig. 3). The center of the projection of the heart on each detector is marked on the patient positioning monitor, allowing for the calculation of the location of the patient’s heart in 3-dimensional space. This will become the center of the cardio-centric orbit. The acquisition can then be started.

##### Acquisition protocols

For rotating detector systems, the main orbit options for cardiac SPECT imaging are body-contoured orbits. IQ-SPECT, however, positions the heart in the center of the collimator field of view and positions the detectors at a 28-cm radius for the cardio-centric orbit. IQ-SPECT uses the flexibility of the gantry to position each detector at an optimal distance to maximize sensitivity gain. Because of the anterior position of the heart in the left hemi-thorax, the fixed angular sample range is in total 208°, from 59° right anterior oblique (RAO) to − 45° left posterior oblique (LPO), what covers the usual 180° in standard SPECT plus the two-fold fan angles of the collimator. For current SPECT imaging system, the imaging resolution is between 13 and 16 mm. The standard matrix size is 128 × 128 pixel, with a zoom factor of 1.00. The most commonly used acquisition mode of IQ-SPECT systems is the “step-and-shoot” method. In this approach, the camera acquires a projection but interrupts data recording during rotation to the next angle. For ^99m^Tc and ^201^Tl, 17 views per head over 104° are recommended (Hawman 2012). There is a minimum requirement of 9 cts/pixel in the posterolateral wall in projection 17 or 18 in order to achieve a useful image quality. In addition, a minimum projection time of 9 s is recommended. Shorter projection reduces the averaging effect across the respiratory cycle. A 9-s time per projection results in a total acquisition time of about 4 min. However, increasing acquisition times up to 10 min allows to reduce injected activity and is recommended for the first low-dose acquisition of a 1-day protocol. Due to the magnification factor, IQ-SPECT is more sensitive to motion artifacts compared to conventional SPECT systems.

The cardiac cycle is divided into frames representing different phases of the cardiac cycle. Using ECG-gated IQ-SPECT, the heartbeat is usually divided into 8 or 16 temporal frames or gates. The R-wave of the QRS complex serves as the signal and starting point (triggering point) of the cardiac cycle. Ideally, the length of acquisition (expressed in seconds per projection) for a gated ^99m^Tc SPECT study does not exceed the traditionally necessary time for a non-gated SPECT study. Time per projection must be adjusted to obtain an adequate myocardial count rate per interval. In general, a total acquisition time between 4 and 10 min (depending on heart rhythm and injected activity) results in adequate image quality of gated acquisitions. This range is mainly based on the preferred balance of injected dose and acquisition time: the lower the dose, the longer the scanning time; the higher the dose, the shorter the acquisition time.

For hybrid imaging systems, the CT configuration can be a low-resolution CT (non-diagnostic CT) or a multidetector-row CT with slices ranging from 2 up to 16. Any of these systems can be used for attenuation correction of MPS. For CACS, at least a 4-slice CT is required (≥ 6 slice recommended). For CCTA, at least a 16-slice scanner is required (≥ 64-slice multidetector-row CT recommended), with imaging capability for slice width of 0.4–0.6 mm and temporal resolution of 500 ms or less (≤ 350 ms is preferred).

##### Quality controls

At the end of the acquisition, the quality of ECG gating, centering, and motion should be checked (Fig. 3). Since the acquisition duration is short, the acquisition can be easily repeated if required (the CT part does not need to be repeated).

##### Image reconstruction

After data acquisition is complete the study is transferred to the “Symbia.net First User” workstation for reconstruction (Table 2). IQ-SPECT uses a conjugate gradient algorithm reconstruction. The projection data should be reviewed for motion; motion correction should be applied in the vertical direction, if necessary (Fig. 3).

**Table 2:** Recommended reconstruction parameters IQ-SPECT

^99m^Tc data	^201^Tl data
Gated reconstruction parameters:	Gated reconstruction parameters:
12 iterations	12 iterations
1 subset	1 subset
10-mm Gaussian smooth (adjust as needed)	7–10-mm Gaussian smooth (adjust as needed). No scatter correction.
Approximate reconstruction times:	Approximate reconstruction times:
8 time bins → 1.5 min	8 time bins → 1.5 min
16 time bins → 3.0 min	16 time bins → 3.0 min
Non-gated AC and No AC reconstruction parameters:	Non-gated AC and No AC reconstruction parameters:
10 iterations	10 iterations
3 subsets	3 subsets
10-mm Gaussian smooth (adjust as needed)	7–10-mm Gaussian smooth (adjust as needed). No scatter correction
Approximate reconstruction time:	Approximate reconstruction time:
Less than 1 min for single non-gated data set, AC and NC	Less than 1 min for single non-gated data set, AC and NC

Gated reconstructions with 8 time bins are completed within 90 s, and non-gated reconstructions are completed in less than 60 s. The reconstructed gated dataset can be loaded into standard commercially available software programs. Segmentation, semi-quantitative analysis, and visual scoring are performed according to the standard recommendation (Cerqueira et al. 2002). IQ-SPECT produces systematically smaller LV volumes than the conventional LEHR MPS protocols and volume estimates are also software dependent (Hippelainen et al. 2017). The calculated LVEF may differ between conventional gated SPECT and gated IQ-SPECT depending on the software program used (Yoneyama et al. 2017; Joergensen and Hansson 2015). In general, IQ-SPECT shows higher values. IQ-SPECT normal databases for 4 min ^99m^Tc acquisitions are available in Cedars 2009 and Corridor 4DM 6.1.5.

##### Artifacts and pitfalls

Prone imaging has been reported to reduce patient motion and attenuation of the inferior wall compared to supine imaging (Takamura et al. 2015)*.* When no ECG gating and no CTAC is performed, the combination of supine and prone images may be helpful. With this approach, attenuation artifacts due to breast and/or excessive lateral chest wall fat can be identified due to the shift in the position of the attenuating structures that occur between the two imaging positions (i.e., prone vs. supine). Prone imaging does not eliminate attenuation artifacts, but simply changes its location. By comparing supine and prone images, artefactual defects will change their location, whereas true perfusion defects will remain fixed (Takamura et al. 2015). It is important that comparison of the rest and stress studies is done with the patient in the same position, especially in NAC reconstructions. IQ-SPECT needs to be acquired including AC, because attenuation artifacts are more marked than with parallel collimation and are also position-dependent, making AC mandatory. Users of a Symbia camera not equipped with a CT scanner who are considering installing an IQ-SPECT system should be aware of this limitation and pay utmost attention to exact reproducible positioning for rest and stress as well as using the combination of supine and prone imaging in all questionable cases*.*

Ideally, images should be compared to gender-balanced ^99m^Tc-sestamibi and ^99m^Tc-tetrofosmin IQ-SPECT normal databases. Typical normal myocardial perfusion distribution with ^99m^Tc tracers in the supine position shows relatively low (i.e., attenuated) myocardial counts in the inferior and inferolateral walls. Tissue attenuation counts are usually more pronounced in the inferior wall in males than in females. Prone imaging compensates for these attenuated inferior myocardial counts to some extent. Although AC compensated for inferior low myocardial counts, low counts at the apex were observed (Nakajima et al. 2017). When the myocardial perfusion distributions of IQ-SPECT ^99m^Tc and ^201^Tl normal databases were visually compared, they showed a similar pattern. However, attenuation-corrected myocardial counts at the apex were lower in ^201^Tl supine imaging than in ^99m^Tc supine imaging (Nakajima et al. 2017). If an abnormality is seen only on the AC images while the NAC images look normal, an AC artifact is then very likely (e.g., due to misregistration of CT and SPECT images since alignment cannot always be perfect or due to a notch artifact on the left margin of the heart, a finding that is not uncommon with single-slice or two-slice CT scans). If a nonreversible apical thinning pattern is seen (a frequent finding), look carefully at the gated images since evidence of akinesis, or at least hypokinesis, of the apex can lead to the (rare) conclusion that a small non-transmural or transmural apical scar is present. Similarly, if the apical thinning pattern is partially reversible, an artifact is likely. More generally, as a rule of thumb, if abnormalities are seen on AC images, always cross-check the AC images with the NAC images. If the abnormalities are real, a faint similar trend should at least be observed (Gremillet and Agostini 2016).

#### Clinical validation

##### Myocardial perfusion images with IQ-SPECT vs. conventional gamma cameras

Pirich et al. (2017) compared IQ-SPECT with conventional LEHR SPECT imaging in 80 patients suspected of CAD. They found no significant difference in perfusion abnormalities between both techniques using the 17-segment scoring analysis method (SSS, SRS, SDS). LVEF assessment was 8% lower with gated IQ-SPECT against conventional LEHR SPECT. Matsuo et al. (2015) compared in 40 low-likelihood normal patients the aspects of stress-rest MPS with ^201^Tl acquired with LEHR SPECT and the IQ-SPECT with or without X-ray CT-derived attenuation correction. The quality of MPS acquired in a shorter time using the IQ-SPECT was equivalent to that of conventional LEHR SPECT. CT-based attenuation correction of MPS acquired with the IQ-SPECT resulted into less attenuation artifacts in the inferior wall. Nakajima et al. (2017) compared in 116 patients suspected of having ischemic heart disease the results of stress-rest MPS with ^99m^Tc-tetrofosmin acquired with LEHR SPECT and the IQ-SPECT in supine and prone positions. Twenty-six patients (22%) showed myocardial ischemia. The discrepancy rate between MPS with IQ-SPECT and LEHR SPECT-CT was low (1.7%) for all coronary territories with a slightly lower discrepancy rate for IQ-SPECT acquired in prone than in supine position.

##### Diagnostic performance in comparison to conventional gamma cameras

A total of 36 patients underwent adenosine stress-rest MPS with ^201^Tl (Konishi et al. 2017). Images were acquired with IQ-SPECT in one quarter of the standard time required for conventional SPECT. Sensitivity, specificity, and accuracy for the detection of CAD were 88.5%, 86.8%, and 87.3% for IQ-SPECT with CTAC including scatter correction and 80.8%, 78.9%, and 79.4% for the conventional SPECT, respectively. The diagnostic value of IQ-SPECT prone imaging was also examined in 129 consecutive patients with suspected ischemic heart disease who underwent IQ-SPECT in the prone position as gold standard with the presence of coronary stenosis > 75% on invasive angiography (Nakajima et al. 2017). The sensitivity, specificity, and accuracy for the detection of significant coronary stenosis were 70%, 90%, and 80%, respectively. Specificity in the RCA territory was found similar to other coronary territories suggesting only a limited impact of attenuation artifacts in the inferior wall in the prone position using the IQ-SPECT.

##### Prognostic value

No data has been published so far on the prognostic value of MPS acquired with the IQ-SPECT.

## Radiation exposure

The dramatic increase in the detection of myocardial counts using cardiac-centered cameras allows for a reduction in activities of perfusion tracers injected into patients. The choice of injected activities of perfusion tracer to be administered to patients is a compromise between image quality and radiation exposure and depends on the patient characteristics (body weight, age), the choice of radiopharmaceutical (^99m^Tc-compounds or ^201^Tl), the presence or absence of coronary artery disease, and previous myocardial infarction and the acquisition protocols (1- or 2-day protocols, imaging time). ^99m^Tc agents should be preferred over ^201^Tl because of their shorter half-life, significantly lower effective dose, and superior image quality. For 1-day protocols, it is recommended to start with the stress acquisition. If the stress MPS results are normal, the rest scan can be omitted, with significant savings in cost, time, and radiotracer exposure to the patient (34). For MPS using the D-SPECT, the activities injected to patients can be reduced by about 30–50% in comparison to the usual recommended activities with Anger gamma cameras resulting into a reduction of the same level in patient radiation exposure. The average total radiation exposure of a stress/rest perfusion study with recommended injected activities for cardiac-centered cameras is approximatively 6–9 mSv with ^99m^Tc-radiolabeled perfusion tracers and 10–15 mSv with ^201^Tl. In addition, ultra-low dose protocols with ^99m^Tc-labeled perfusion tracers allow for an additional decrease in patient radiation exposure down to 1–2 mSv for stress-only acquisitions and 4–6 mSv for stress/rest acquisitions (Einstein et al. 2014; Songy et al. 2018). This ultra-low dose protocols should be preferred in young individuals with a low likelihood of CAD.

## References

[CR1] Agostini D, Marie PY, Ben-Haim S, Rouzet F, Songy B, Giordano A (2016). Performance of cardiac cadmium-zinc-telluride gamma camera imaging in coronary artery disease: a review from the cardiovascular committee of the European Association of Nuclear Medicine (EANM). Eur J Nucl Med Mol Imaging.

[CR2] Allie R, Hutton BF, Prvulovich E, Bomanji J, Michopoulou S, Ben-Haim S (2016). Pitfalls and artifacts using the D-SPECT dedicated cardiac camera. J Nucl Cardiol.

[CR3] Barone-Rochette G, Zoreka F, Djaileb L, Piliero N, Calizzano A, Quesada JL et al (2018) Diagnostic value of stress thallium-201/rest technetium-99m-sestamibi sequential dual isotope high-speed myocardial perfusion imaging for the detection of haemodynamically relevant coronary artery stenosis. J Nucl Cardiol 10.1007/s12350-018-1189-8 10.1007/s12350-018-1189-8 29380286

[CR4] Ben-Haim S, Almukhailed O, Neill J, Slomka P, Allie R, Shiti D (2014). Clinical value of supine and upright myocardial perfusion imaging in obese patients using the D-SPECT camera. J Nucl Cardiol.

[CR5] Ben-Haim S, Kacperski K, Hain S, Van Gramberg D, Hutton BF, Erlandsson K (2010). Simultaneous dual-radionuclide myocardial perfusion imaging with a solid-state dedicated cardiac camera. Eur J Nucl Med Mol Imaging.

[CR6] Berman DS, Kang X, Tamarappoo B, Wolak A, Hayes SW, Nakazato R (2009). Stress thallium-201/rest technetium-99m sequential dual isotope high-speed myocardial perfusion imaging. JACC Cardiovasc Imaging.

[CR7] Buechel RR, Herzog BA, Husmann L, Burger IA, Pazhenkottil AP, Treyer V (2010). Ultrafast nuclear myocardial perfusion imaging on a new gamma camera with semiconductor detector technique: first clinical validation. Eur J Nucl Med Mol Imaging.

[CR8] Caobelli F, Akin M, Thackeray JT, Brunkhorst T, Widder J, Berding G (2016). Diagnostic accuracy of cadmium-zinc-telluride-based myocardial perfusion SPECT: impact of attenuation correction using a co-registered external computed tomography. Eur Heart J Cardiovasc Imaging.

[CR9] Cerqueira MD, Weissman NJ, Dilsizian V, Jacobs AK, Kaul S, Laskey WK (2002). Standardized myocardial segmentation and nomenclature for tomographic imaging of the heart. A statement for healthcare professionals from the cardiac imaging Committee of the Council on clinical cardiology of the American Heart Association. Int J Cardiovasc Imaging.

[CR10] Chowdhury FU, Vaidyanathan S, Bould M, Marsh J, Trickett C, Dodds K (2014). Rapid-acquisition myocardial perfusion scintigraphy (MPS) on a novel gamma camera using multipinhole collimation and miniaturized cadmium-zinc-telluride (CZT) detectors: prognostic value and diagnostic accuracy in a ‘real-world’ nuclear cardiology service. Eur Heart J Cardiovasc Imaging.

[CR11] DePuey EG (2012). Advances in SPECT camera software and hardware: currently available and new on the horizon. J Nucl Cardiol.

[CR12] Einstein AJ, Blankstein R, Andrews H, Fish M, Padgett R, Hayes SW (2014). Comparison of image quality, myocardial perfusion, and left ventricular function between standard imaging and single-injection ultra-low-dose imaging using a high-efficiency SPECT camera: the MILLISIEVERT study. J Nucl Med.

[CR13] Engbers EM, Timmer JR, Mouden M, Knollema S, Jager PL, Ottervanger JP (2017). Prognostic value of myocardial perfusion imaging with a cadmium-zinc-telluride SPECT camera in patients suspected of having coronary artery disease. J Nucl Med.

[CR14] Erlandsson K, Kacperski K, van Gramberg D, Hutton BF (2009). Performance evaluation of D-SPECT: a novel SPECT system for nuclear cardiology. Phys Med Biol.

[CR15] Esteves FP, Galt JR, Folks RD, Verdes L, Garcia EV (2014). Diagnostic performance of low-dose rest/stress Tc-99m tetrofosmin myocardial perfusion SPECT using the 530c CZT camera: quantitative vs visual analysis. J Nucl Cardiol.

[CR16] Esteves FP, Raggi P, Folks RD, Keidar Z, Askew JW, Rispler S (2009). Novel solid-state-detector dedicated cardiac camera for fast myocardial perfusion imaging: multicenter comparison with standard dual detector cameras. J Nucl Cardiol.

[CR17] Fiechter M, Gebhard C, Fuchs TA, Ghadri JR, Stehli J, Kazakauskaite E (2012). Cadmium-zinc-telluride myocardial perfusion imaging in obese patients. J Nucl Med.

[CR18] Fiechter M, Ghadri JR, Kuest SM, Pazhenkottil AP, Wolfrum M, Nkoulou RN (2011). Nuclear myocardial perfusion imaging with a novel cadmium-zinc-telluride detector SPECT/CT device: first validation versus invasive coronary angiography. Eur J Nucl Med Mol Imaging.

[CR19] Gambhir SS, Berman DS, Ziffer J, Nagler M, Sandler M, Patton J (2009). A novel high-sensitivity rapid-acquisition single-photon cardiac imaging camera. J Nucl Med.

[CR20] Gimelli A, Achenbach S, Buechel RR, Edvardsen T, Francone M, Gaemperli O (2018). Strategies for radiation dose reduction in nuclear cardiology and cardiac computed tomography imaging: a report from the European Association of Cardiovascular Imaging (EACVI), the cardiovascular Committee of European Association of nuclear medicine (EANM), and the European Society of Cardiovascular Radiology (ESCR). Eur Heart J.

[CR21] Gimelli A, Bottai M, Giorgetti A, Genovesi D, Kusch A, Ripoli A (2011). Comparison between ultrafast and standard single-photon emission CT in patients with coronary artery disease: a pilot study. Circ Cardiovasc Imaging.

[CR22] Gimelli A, Liga R, Duce V, Kusch A, Clemente A, Marzullo P (2017). Accuracy of myocardial perfusion imaging in detecting multivessel coronary artery disease: a cardiac CZT study. J Nucl Cardiol.

[CR23] Giorgetti A, Masci PG, Marras G, Rustamova YK, Gimelli A, Genovesi D (2013). Gated SPECT evaluation of left ventricular function using a CZT camera and a fast low-dose clinical protocol: comparison to cardiac magnetic resonance imaging. Eur J Nucl Med Mol Imaging.

[CR24] Goto K, Takebayashi H, Kihara Y, Yamane H, Hagikura A, Morimoto Y (2014). Impact of combined supine and prone myocardial perfusion imaging using an ultrafast cardiac gamma camera for detection of inferolateral coronary artery disease. Int J Cardiol.

[CR25] GP HP (2012). IQ.SPECT: a technical and clinical overview. White paper. Siemens medical solutions, USA molecular imaging.

[CR26] Gremillet E, Agostini D (2016). How to use cardiac IQ*SPECT routinely? An overview of tips and tricks from practical experience to the literature. Eur J Nucl Med Mol Imaging.

[CR27] Herzog BA, Buechel RR, Husmann L, Pazhenkottil AP, Burger IA, Wolfrum M (2010). Validation of CT attenuation correction for high-speed myocardial perfusion imaging using a novel cadmium-zinc-telluride detector technique. J Nucl Med.

[CR28] Hesse B, Tagil K, Cuocolo A, Anagnostopoulos C, Bardies M, Bax J (2005). EANM/ESC procedural guidelines for myocardial perfusion imaging in nuclear cardiology. Eur J Nucl Med Mol Imaging.

[CR29] Hippelainen E, Makela T, Kaasalainen T, Kaleva E (2017). Ejection fraction in myocardial perfusion imaging assessed with a dynamic phantom: comparison between IQ-SPECT and LEHR. EJNMMI Phys.

[CR30] Hudson HM, Larkin RS (1994). Accelerated image reconstruction using ordered subsets of projection data. IEEE Trans Med Imaging.

[CR31] Imbert L, Marie PY (2016). CZT cameras: a technological jump for myocardial perfusion SPECT. J Nucl Cardiol.

[CR32] Imbert L, Poussier S, Franken PR, Songy B, Verger A, Morel O (2012). Compared performance of high-sensitivity cameras dedicated to myocardial perfusion SPECT: a comprehensive analysis of phantom and human images. J Nucl Med.

[CR33] Joergensen T, Hansson SH (2015). Evaluation of the left ventricular ejection fraction with gated IQ-SPECT myocardial perfusion imaging. J Nucl Med Technol..

[CR34] Kincl V, Kaminek M, Vasina J, Panovsky R, Havel M (2016). Feasibility of ultra low-dose thallium stress-redistribution protocol including prone imaging in obese patients using CZT camera. Int J Cardiovasc Imaging..

[CR35] Konishi T, Nakajima K, Okuda K, Yoneyama H, Matsuo S, Shibutani T (2017). IQ-SPECT for thallium-201 myocardial perfusion imaging: effect of normal databases on quantification. Ann Nucl Med.

[CR36] Liu CJ, Cheng JS, Chen YC, Huang YH, Yen RF (2015). A performance comparison of novel cadmium-zinc-telluride camera and conventional SPECT/CT using anthropomorphic torso phantom and water bags to simulate soft tissue and breast attenuation. Ann Nucl Med.

[CR37] Matsuo S, Nakajima K, Onoguchi M, Wakabayash H, Okuda K, Kinuya S (2015). Nuclear myocardial perfusion imaging using thallium-201 with a novel multifocal collimator SPECT/CT: IQ-SPECT versus conventional protocols in normal subjects. Ann Nucl Med.

[CR38] Meyer C, Weinmann P (2017). Validation of early image acquisitions following Tc-99 m sestamibi injection using a semiconductors camera of cadmium-zinc-telluride. J Nucl Cardiol.

[CR39] Mouden M, Timmer JR, Ottervanger JP, Reiffers S, Oostdijk AH, Knollema S (2012). Impact of a new ultrafast CZT SPECT camera for myocardial perfusion imaging: fewer equivocal results and lower radiation dose. Eur J Nucl Med Mol Imaging.

[CR40] Nakajima K, Okuda K, Momose M, Matsuo S, Kondo C, Sarai M (2017). IQ.SPECT technology and its clinical applications using multicenter normal databases. Ann Nucl Med.

[CR41] Nakazato R, Berman DS, Hayes SW, Fish M, Padgett R, Xu Y (2013). Myocardial perfusion imaging with a solid-state camera: simulation of a very low dose imaging protocol. J Nucl Med.

[CR42] Nakazato R, Tamarappoo BK, Kang X, Wolak A, Kite F, Hayes SW (2010). Quantitative upright-supine high-speed SPECT myocardial perfusion imaging for detection of coronary artery disease: correlation with invasive coronary angiography. J Nucl Med.

[CR43] Neill J, Prvulovich EM, Fish MB, Berman DS, Slomka PJ, Sharir T (2013). Initial multicentre experience of high-speed myocardial perfusion imaging: comparison between high-speed and conventional single-photon emission computed tomography with angiographic validation. Eur J Nucl Med Mol Imaging.

[CR44] Nishiyama Y, Miyagawa M, Kawaguchi N, Nakamura M, Kido T, Kurata A (2014). Combined supine and prone myocardial perfusion single-photon emission computed tomography with a cadmium zinc telluride camera for detection of coronary artery disease. Circ J.

[CR45] Nkoulou R, Pazhenkottil AP, Kuest SM, Ghadri JR, Wolfrum M, Husmann L (2011). Semiconductor detectors allow low-dose-low-dose 1-day SPECT myocardial perfusion imaging. J Nucl Med.

[CR46] Nudi F, Iskandrian AE, Schillaci O, Peruzzi M, Frati G, Biondi-Zoccai G (2017). Diagnostic accuracy of myocardial perfusion imaging with CZT technology: systemic review and meta-analysis of comparison with invasive coronary angiography. JACC Cardiovasc Imaging.

[CR47] Oddstig J, Martinsson E, Jogi J, Engblom H, Hindorf C (2018) Differences in attenuation pattern in myocardial SPECT between CZT and conventional gamma cameras. J Nucl Cardiol 10.1007/s12350-018-1296-6 10.1007/s12350-018-1296-6 PMC6908561 29796975

[CR48] Oldan JD, Shaw LK, Hofmann P, Phelan M, Nelson J, Pagnanelli R (2016). Prognostic value of the cadmium-zinc-telluride camera: a comparison with a conventional (anger) camera. J Nucl Cardiol.

[CR49] Perrin M, Djaballah W, Moulin F, Claudin M, Veran N, Imbert L (2015). Stress-first protocol for myocardial perfusion SPECT imaging with semiconductor cameras: high diagnostic performances with significant reduction in patient radiation doses. Eur J Nucl Med Mol Imaging.

[CR50] Pirich C, Keinrath P, Barth G, Rendl G, Rettenbacher L, Rodrigues M (2017). Diagnostic accuracy and functional parameters of myocardial perfusion scintigraphy using accelerated cardiac acquisition with IQ SPECT technique in comparison to conventional imaging. Q J Nucl Med Mol Imaging.

[CR51] Sharir T, Pinskiy M, Pardes A, Rochman A, Prokhorov V, Kovalski G (2016). Comparison of the diagnostic accuracies of very low stress-dose with standard-dose myocardial perfusion imaging: automated quantification of one-day, stress-first SPECT using a CZT camera. J Nucl Cardiol.

[CR52] Sharir T, Slomka PJ, Hayes SW, DiCarli MF, Ziffer JA, Martin WH (2010). Multicenter trial of high-speed versus conventional single-photon emission computed tomography imaging: quantitative results of myocardial perfusion and left ventricular function. J Am Coll Cardiol.

[CR53] Songy B, Guernou M, Hivoux D, Attias D, Lussato D, Queneau M (2018). Prognostic value of one millisievert exercise myocardial perfusion imaging in patients without known coronary artery disease. J Nucl Cardiol.

[CR54] Songy B, Guernou M, Lussato D, Queneau M, Geronazzo R (2012). Low-dose thallium-201 protocol with a cadmium-zinc-telluride cardiac camera. Nucl Med Commun.

[CR55] Takahashi Y, Miyagawa M, Nishiyama Y, Ishimura H, Mochizuki T (2013). Performance of a semiconductor SPECT system: comparison with a conventional anger-type SPECT instrument. Ann Nucl Med.

[CR56] Takamura T, Horiguchi Y, Kanna M, Matsushita H, Sudo Y, Kikuchi S (2015). Validation of prone myocardial perfusion SPECT with a variable-focus collimator versus supine myocardial perfusion SPECT with or without computed tomography-derived attenuation correction. Ann Nucl Med.

[CR57] Verberne HJ, Acampa W, Anagnostopoulos C, Ballinger J, Bengel F, De Bondt P (2015). EANM procedural guidelines for radionuclide myocardial perfusion imaging with SPECT and SPECT/CT: 2015 revision. Eur J Nucl Med Mol Imaging.

[CR58] Verger A, Djaballah W, Fourquet N, Rouzet F, Koehl G, Imbert L (2013). Comparison between stress myocardial perfusion SPECT recorded with cadmium-zinc-telluride and anger cameras in various study protocols. Eur J Nucl Med Mol Imaging.

[CR59] Verger A, Imbert L, Yagdigul Y, Fay R, Djaballah W, Rouzet F (2014). Factors affecting the myocardial activity acquired during exercise SPECT with a high-sensitivity cardiac CZT camera as compared with conventional anger camera. Eur J Nucl Med Mol Imaging.

[CR60] Xu Y, Nakazato R, Hayes S, Hachamovitch R, Cheng VY, Gransar H (2011). Prognostic value of automated vs visual analysis for adenosine stress myocardial perfusion SPECT in patients without prior coronary artery disease: a case-control study. J Nucl Cardiol.

[CR61] Yokota S, Mouden M, Ottervanger JP, Engbers E, Knollema S, Timmer JR (2016). Prognostic value of normal stress-only myocardial perfusion imaging: a comparison between conventional and CZT-based SPECT. Eur J Nucl Med Mol Imaging.

[CR62] Yoneyama H, Shibutani T, Konishi T, Mizutani A, Hashimoto R, Onoguchi M (2017). Validation of left ventricular ejection fraction with the IQ*SPECT system in small-heart patients. J Nucl Med Technol.

[CR63] Zoccarato O, Lizio D, Savi A, Indovina L, Scabbio C, Leva L (2016). Comparative analysis of cadmium-zincum-telluride cameras dedicated to myocardial perfusion SPECT: a phantom study. J Nucl Cardiol.

